# Application of Integrative Medicine in Plastic Surgery: A Real-World Data Study

**DOI:** 10.3390/medicina61081405

**Published:** 2025-08-01

**Authors:** David Lysander Freytag, Anja Thronicke, Jacqueline Bastiaanse, Ioannis-Fivos Megas, David Breidung, Ibrahim Güler, Harald Matthes, Sophia Johnson, Friedemann Schad, Gerrit Grieb

**Affiliations:** 1 Department of Plastic Surgery and Hand Surgery, Gemeinschaftskrankenhaus Havelhoehe, Kladower Damm 221, 14089 Berlin, Germany; lysanderfreytag@gmail.com (D.L.F.); jacqueline.bastiaanse@havelhoehe.de (J.B.); ibrahim.gueler@havelhoehe.de (I.G.); 2 Research Institute Havelhöhe, Kladower Damm 221, 14089 Berlin, Germany; anja.thronicke@havelhoehe.de (A.T.); harald.matthes@havelhoehe.de (H.M.); sophia.johnson@uni-jena.de (S.J.); friedemann.schad@havelhoehe.de (F.S.); 3 Department of Orthopaedic and Trauma Surgery, Center of Plastic Surgery, Hand Surgery and Microsurgery, Evangelisches Waldkrankenhaus Spandau, Stadtrandstr. 555, 13589 Berlin, Germany; fivos.megas@gmail.com; 4 Department of Plastic, Reconstructive and Hand Surgery, Burn Center for Severe Burn Injuries, Klinikum Nuremberg Hospital, Paracelsus Medical University, Breslauer Str. 201, 90471 Nuremberg, Germany; david.breidung@icloud.com; 5 Medical Clinic for Gastroenterology, Infectiology and Rheumatology, CBF, Charité University Medicine Berlin, Hindenburgdamm 30, 12200 Berlin, Germany; 6 Interdisciplinary Oncology and Palliative Care, Gemeinschaftskrankenhaus Havelhoehe, Kladower Damm 221, 14089 Berlin, Germany; 7 Department of Plastic Surgery and Hand Surgery, Burn Center, Medical Faculty, RWTH Aachen University, Pauwelsstrasse 30, 52074 Aachen, Germany

**Keywords:** plastic surgery, integrative medicine, anthroposophic medicine

## Abstract

*Background and Objectives*: There is a global rise of public interest in integrative medicine. The principles of integrative medicine combining conventional medicine with evidence-based complementary therapies have been implemented in many medical areas, including plastic surgery, to improve patient’s outcome. The aim of the present study was to systematically analyze the application and use of additional non-pharmacological interventions (NPIs) of patients of a German department of plastic surgery. *Materials and Methods*: The present real-world data study utilized data from the Network Oncology registry between 2016 and 2021. Patients included in this study were at the age of 18 or above, stayed at the department of plastic surgery and received at least one plastic surgical procedure. Adjusted multivariable logistic regression analyses were performed to detect associations between the acceptance of NPIs and predicting factors such as age, gender, year of admission, or length of hospital stay. *Results*: In total, 265 patients were enrolled in the study between January 2016 and December 2021 with a median age of 65 years (IQR: 52–80) and a male/female ratio of 0.77. Most of the patients received reconstructive surgery (90.19%), followed by hand surgery (5.68%) and aesthetic surgery (2.64%). In total, 42.5% of the enrolled patients accepted and applied NPIs. Physiotherapy, rhythmical embrocations, and compresses were the most often administered NPIs. *Conclusions*: This exploratory analysis provides a descriptive overview of the application and acceptance of NPIs in plastic surgery patients within a German integrative care setting. While NPIs appear to be well accepted by a subset of patients, further prospective studies are needed to evaluate their impact on clinical outcomes such as postoperative recovery, pain management, patient-reported quality of life, and overall satisfaction with care.

## 1. Introduction

Following the rise of epidemics and pandemics, lately and most notably, the COVID-19 pandemic, we have seen a global rise in public interest in integrative medicine [1]. Integrative medicine focuses not only on the physical body but also on aspects of the individual mind and soul in the treatment of patients, thereby allowing optimal healing and general improvement of patents’ health-related quality of life [2]. In the broader literature, integrative medicine is used to denote treatment that combines conventional medicine with evidence-based complementary therapies and that stresses the importance of the relationship between the practitioner and the patient to achieve optimal care for each individual [1]. The principles of integrative medicine are already well-known and have been implemented in many medical areas. In oncology, for example, mistletoe therapy is implemented alongside the standard-care therapies to increase tolerance to the toxicity of oncological therapies and to improve health-related quality of life. [3]. In cardiology, rhythmic massages can be used to influence the heart rate variability [4]. The integrative treatment of patients is also adopted in surgery, including plastic surgery, to improve patients’ outcomes [5]. The concept of integrative medicine, especially with respect to anthroposophic medicine, is well-established and in demand by many patients in German-speaking parts of Europe [6]. One of the German proponents of the integrative approach is the hospital Gemeinschaftskrankenhaus Havelhöhe (GKH) in Berlin. Here, the three dimensions of mind, body, and soul are taken into consideration in both diagnosis and therapy. In the Department of Plastic Surgery of the GKH, all inpatients apply non-pharmacological interventions (NPIs) in addition to their operative and standard-of-care pharmacological treatments. The NPIs include breathing therapy, eurythmy, painting therapy, music therapy, massages, physiotherapy, psychotherapy, rhythmic embrocations, compresses, biographical interviews, psychoeducation, and integrated psychosomatics.

Integrative medicine has been increasingly explored in surgical disciplines for its potential to address perioperative anxiety, pain, and recovery [7,8,9,10]. A landmark meta-analysis by Hole in The Lancet demonstrated that music interventions significantly reduced postoperative pain and anxiety, decreased analgesic use, and improved patient satisfaction across a range of surgical procedures [7]. In addition, guided imagery—a cognitive-behavioral technique involving structured mental visualization—has shown benefit in surgical contexts. For example, a randomized controlled trial found that guided imagery significantly reduced postoperative pain following lower extremity surgery [9]. Similarly, perioperative guided imagery significantly reduced anxiety and pain while improving sleep quality and satisfaction with nursing care among surgical patients [10]. These findings underscore the potential role of NPIs in enhancing recovery and patient experience. However, despite such progress in related specialties, plastic surgery remains underrepresented in the integrative medicine research landscape. Our study aims to address this gap by systematically describing the application and acceptance of NPI therapies in a plastic surgery inpatient setting. At the GKH, which combines modern medicine with non-pharmacological medicine, the aim of the treatment is to strengthen the body and soul in a way that aids the naturally regenerative potential of the patient. This entails treatment of the symptoms experienced by the patient as well as the administration of treatments that activate the body’s self-regulating powers, support the immune system, and awaken physical and psychological resources. To meet individual patients’ needs, a wide range of NPI modules can be combined. The aim of the present study was to descriptively analyze the acceptance and use of NPIs at the GKH between 2016 and 2021 in the Department of Plastic Surgery.

## 2. Materials and Methods

### 2.1. Study Design, Description of Study Participants, and Data Source Assessment

The present real-world data study utilizes data from the Network Oncology registry. Patients included in this study were at least 18 years of age, admitted to the Department of Plastic Surgery between 2016 and 2021, with a minimum stay of five days, and receiving at least one plastic surgical treatment. The five-day threshold was chosen based on institutional practice, as it allows sufficient time for NPIs to be offered and administered. A total of 265 patients met these criteria and were included in the analysis. As this was a descriptive and exploratory study using real-world data, no formal sample size calculation was performed. Instead, the study included all patients who met the predefined inclusion criteria and were treated in the department during the observation period from 2016 to 2021.

Patient-relevant data, such as age at admission, gender, and treatment-relevant information, such as surgery type, were retrieved from the NO registry. Some NPIs were only applied to a patient in a single unit, such as a biographic interview, while other NPIs were applied as multiple units, such as massages, compresses, and physiotherapy.

### 2.2. Ethics Approval and Consent to Participate

The study was approved by the ethics committee of the Medical Association Berlin (Eth-27/10). Written informed consent was obtained from all patients prior to the study enrolment. The study complies with the principles of the Declaration of Helsinki.

### 2.3. Statistical Methods

The continuous variables were described as the median with interquartile range (IQR); the categorical variables were summarized as absolute and relative frequencies. Adjusted multivariable logistic regression analyses were performed to detect associations between the application of NPIs and predicting factors such as age, gender, year of admission, or length of hospital stay. All tests were performed two-sided, and all analyses were exploratory. Missing values were estimated using a coefficient based on NPI acceptance rates according to the length of stay. All analyses were conducted using the software R version 4.1.3 10-03-2022).

### 2.4. Plastic Surgery Procedures

Plastic surgery included reconstructive surgery (chronic wounds, extensive skin soft tissue defects, hand surgery, aesthetic operations) as well as burn surgery (acute burns as well as burn complications). The majority of procedures were reconstructive in nature, reflecting the hospital’s clinical focus. Aesthetic and oncological surgeries were also represented, albeit in smaller numbers.

### 2.5. Complementary Procedures

Eurythmy

Eurythmy is practiced as a type of movement therapy, which supports the patient in almost every illness, physically and mentally, as well as the body’s own regenerative powers. The main therapy component of eurythmy is movement to the sound structure of music and language. Here, vowels and consonants, as well as musical notes, intervals, and harmonies, are reproduced as movement and combined in specific sequences that have different effects on body, soul, and spirit. Eurythmy therapists develop patient- and diagnosis-specific exercise and movement routines for the patient to learn with the therapists and later practice independently. Eurythmy’s positive effects can be seen in the regulation of digestion, metabolism, sleep, breath, and body temperature, as well as cancer-related fatigue syndrome, pain syndromes, and depression.

Breathing Therapy

Breathing is the body function that is most intensely connected with all of the levels of the human. Breathing therapy is utilized to influence the heart’s functions, the circulatory system, and the oxygenation and carbon dioxide levels of the body, as well as the ion concentration. In this way, the body’s metabolism is supported.

Painting therapy

Therapists in painting therapy utilize art therapy to facilitate changes on a body, mind, and soul level that support the healing process of the patient. Here, patients are encouraged to relate visual expression to inner experiences, such as a disease process, and thus release creative energy that brings about change in the patient. Certain painting techniques are more supportive when dealing with specific complaints. For example, the wet-in-wet watercolor technique, with its flowing gradients and soft transitions, is used to support emotional processing and reduce psychological rigidity or inner tension.

Music therapy

Music therapy is used to regulate emotions such as stress, angst, exhaustion, or depression, as well as treat symptoms resulting from chronic or postoperative pain, nausea, dizziness, or disturbances to the vital rhythms, such as insomnia. Music therapy is characterized by a patient-specific therapeutic exercise regimen. This can manifest in an active manner, such as through the playing of specific instruments in combination with singing and movement, or a receptive manner, such as listening to and experiencing specific music and acoustic harmonies. These rhythmical repetitions can support the regulation and coordination of autonomic and physiological processes, thereby improving overall physical well-being.

Massages

Massages are also employed in the treatment processes. In rhythmical alternation, the lower calf and back region and the upper back, neck, and arm region are massaged to encourage harmony between the metabolism and the nervous system. In this way, the body’s ability to self-regulate is increased. Not only does this process loosen hardened muscles but it also harmonizes the functions of the inner organs, dissolves tensions and blockades on the spiritual level, and gives patients a new understanding of the agency they hold over their body.

Rhythmical embrocations

Similar to massage, rhythmical embrocations also involve rhythmical touches to the body. However, in embrocations, the therapist uses light stroking movements that either gently compress or loosen the body. These movements affect the body on a deeper level and bring the body fluids into movement. Blockades, deposits, and firmnesses start to flow, the breath becomes deeper, and pain and tension can be loosened. These embrocations provide a sustained strengthening of the body’s own rhythms. As a result, things such as sleep quality, blood pressure, and metabolism are improved.

Compresses

Compresses are frequently applied with the aim of promoting coherence between physical and emotional states and initiating healing processes from within. In practical terms, compresses consist of three layers of cloth enveloping the patient and targeting the issue area with a poultice in the first layer closest to the skin. The poultice is composed of a number of natural ingredients and varies depending on the area targeted in the body, as well as the specific desired function of the poultice. Essential oils and essences are also typically included.

Physiotherapy

The goal of physiotherapy is to bring the body back to a functional, mobile status. Physiotherapists guide patients through exercises that stimulate physical movement and simultaneously support emotional engagement and well-being. The results are improvements in pain and mental ill-being, and damage and diseases of the locomotor apparatus are reduced. The following physiotherapeutic techniques were used: manual therapy, the Cyriax method, the Bobath concept, sensory integration therapy, sports physiotherapy, medical training therapy, functional movement training, reflective breathing therapy, back school, pelvic floor training, and prosthesis training.

Biographical interviews

Biographical interviews are typically conducted as part of psycho-oncological care. Given the significant psychological and physical burden associated with a cancer diagnosis, patients are offered the opportunity to participate in these biographical interviews as a component of their psychotherapeutic support. In such an interview, the patient and therapist together reflect and discuss the different aspects of the patient’s life that are deeply affected by the cancer diagnosis, such as dreams, goals, and needs that the patient had identified prior to receiving the diagnosis. By asking the patient the question of what seems important post-diagnosis, the interview aims to trigger new impulses within the patient that bring inner peace with the situation at hand.

Psychoeducation and Integrated psychosomatics

Psychoeducation is offered especially to patients with chronic diseases, with the goal of giving them knowledge about their illness as well as methods of coping with living with such an illness.

Integrated psychosomatics are also offered, in which the effects of the mental and spiritual status of the patients on their physical body are examined. Together with the therapists, patients examine and discover connections between their physical and spiritual state and find ways to address any blockages or hindrances to healing. The goal is to achieve better harmony between these three aspects to facilitate a better healing process as well as prevent future poor health.

## 3. Results

### 3.1. Baseline Characteristics

**Figure 1:**
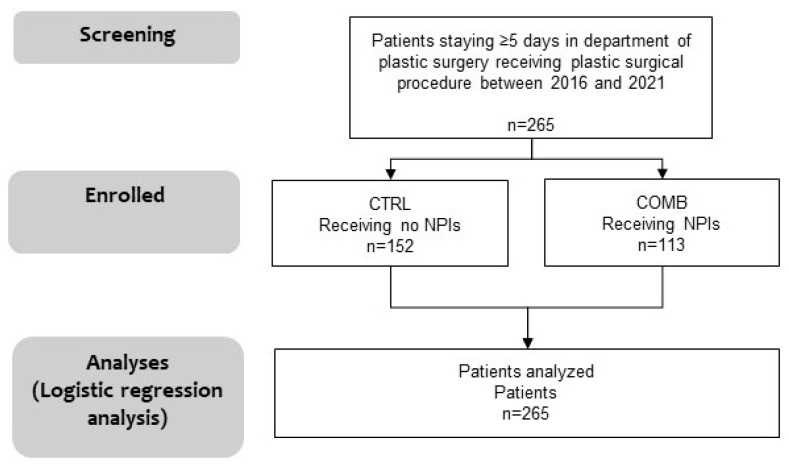
Flow chart of the study. NPI, non-pharmacological intervention; CTRL, patients not receiving NPIs; COMB, patients receiving additional NPIs.

In total, 265 patients were enrolled in the study between January 2016 and December 2021 (see flowchart in Figure 1).

The baseline characteristics of the patients are indicated in Table 1. The age of the enrolled patients ranged from 18 to 102 years, with a median age of 65 years (IQR: 52–80) (Table 1). In total, 56.6% of the participants were female and 43.4% were male (Table 1). The male/female ratio was 0.77.

### 3.2. Plastic Surgery

**Figure 2:**
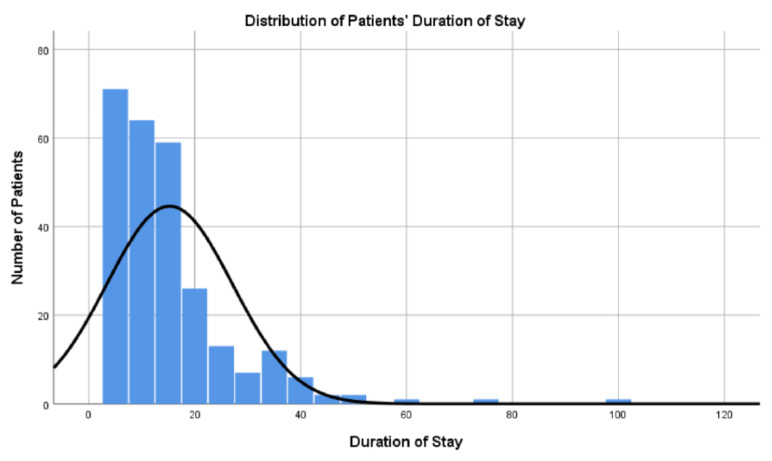
Distribution of patients’ stay.

During the course of their hospital stay, the total cohort of patients received the following types of plastic surgery: reconstructive surgery (90.6%) including reconstructive aesthetic surgery, hand surgery (5.6%), aesthetic surgery (2.6%), oncological surgery (0.4%), and burn surgery (0.8%) including reconstructive burn surgery (Table 2). The median duration of stay per patient was 12 days, with a minimum of 5 days and a maximum of 100 days (Figure 2).

### 3.3. Non-Pharmacological Therapies

**Figure 3:**
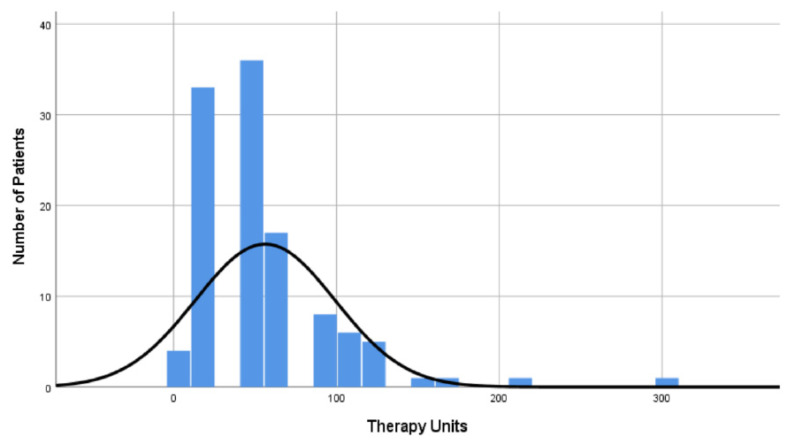
Distribution of NPI units per patient. NPI, non-pharmacological intervention.

In total, 42.5% of the patients received NPIs with a median of 45 applied NPI units (IQR 24-66) and a minimum of 3 and a maximum of 297 NPI units (Figure 3).

**Figure 4:**
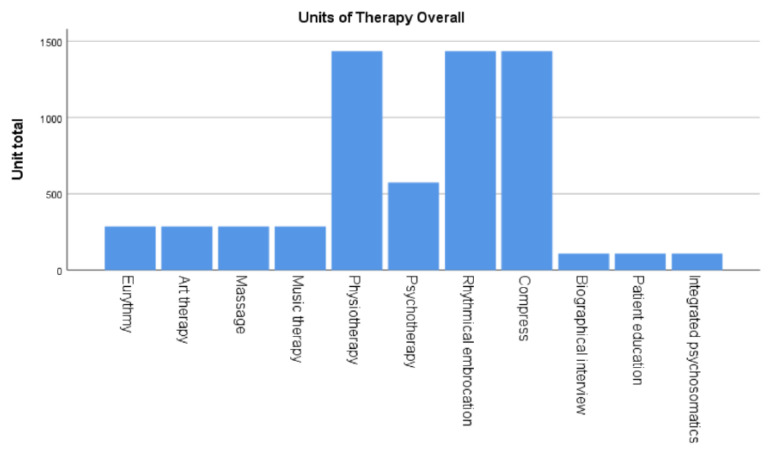
Accumulated number of therapy units per unit.

Physiotherapy, rhythmical embrocations, and compresses were the most often applied NPIs with 1435 total administered units per therapy type (Figure 4). Psychotherapy was the next most administered treatment with 574 units administered in total, followed by eurythmy, art therapy, massage, and music therapy with 287 units administered each. The second least administered therapies were the biographical interview, patient education, and integrated psychosomatics, which were all administered in 109 units (Figure 4).

Almost all NPIs were equally utilized by the patients, with a total of 111 patients (41.9%) receiving at least one unit. Therapies such as patient education or integrated psychosomatics were less applied, with 109 patients (41.1%) receiving these treatments.

On average, 42.6% (between 27.5 and 71.4%) of plastic surgery patients who stayed five days or longer received NPIs in our center (Table 3). There was no clear trend in the number of patients receiving NPIs throughout the years observed.

**Figure 5:**
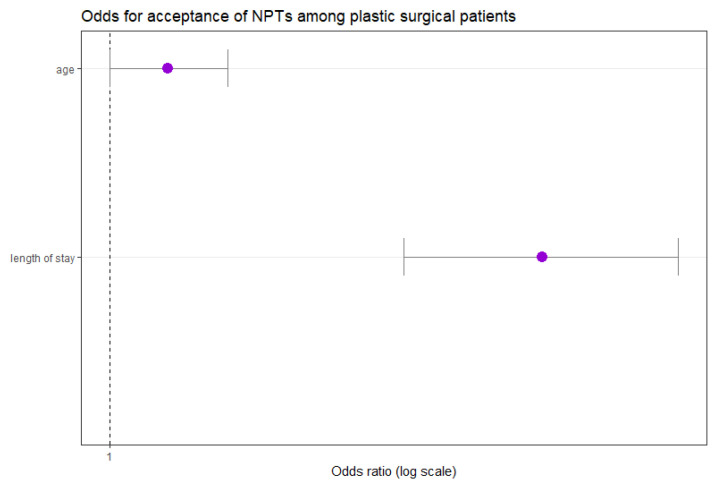
LOS is associated with NPI application among plastic surgical patients. NPIs, non-pharmacological interventions.

To analyze the association between the length of stay (LOS) and the patient’s application of NPIs, we applied adjusted multivariate logistic regression methods. For each additional day of the patient’s stay, the odds of receiving at least one unit of NPI significantly increased by a factor of 1.13 (OR 1.126, 95%CI: 1.084–1.169, *p* < 0.001) (Figure 5). Thus, the probability of a patient receiving at least one NPI increased with longer hospital stays when maintaining gender, age, and year of admission constant.

The age of the patients was not significantly associated with NPI application when adjusting for gender and duration of stay (OR 1.016, 95% CI: 1.000–1.033, *p* = 0.047) (see Figure 5). Furthermore, no statistically significant relationships were observed between the year of admittance and the number of therapy units applied, nor were any sex differences registered.

## 4. Discussion

The demand for integrative therapies in plastic surgery has been noted for decades, but despite their potential economic benefits and patient satisfaction, their adoption in surgical specialties, including plastic surgery, remains limited [6]. Despite sparse current data, there is consensus on the positive effects of these therapies on patients’ mental stress and procedural pain [11]. While non-pharmacological integrative interventions are popular among elective surgery patients, particularly in aesthetic procedures where psychosocial issues are prevalent, formal studies integrating anthroposophic medicine in plastic surgery remain scarce. Further research is, therefore, needed to establish guidelines and assess the efficacy and safety of these approaches in surgical settings. The present study is one of the first that systematically examines the pattern of application of integrative anthroposophic non-pharmacological interventions in a population of plastic surgery patients during a follow-up of 6 years. The findings of our study reveal that between 27.5 and 71.4% of patients accepted NPIs in addition to standard treatment in a plastic surgery department during the years 2015–2021. This is in line with another survey showing that up to 80% of plastic surgery patients apply integrative therapies, including natural products or mind–body practices [12]. A systematic review by Ruan et al. revealed that 61.5% of studies on natural products and 87.5% of studies on mind–body practices revealed a beneficial effect for plastic surgery patients [5]. The fact that, on average, 42% of patients received NPIs may reflect individual patient preferences, clinical condition, or logistic limitations in the scheduling or availability of therapists during the hospital stay. This aligns with broader trends showing that nearly half of surgical patients report complementary treatment use preoperatively, and patient-driven demand for integrative care, including non-pharmacological, whole-person therapies, is on the rise, especially in response to the limitations of conventional medicine [13]. The described patient population of plastic surgery patients included cases for reconstructive surgery (chronic wounds, extensive skin soft tissue defects, hand surgery, aesthetic operations) as well as burn surgery (acute burns as well as burn complications). The heterogeneity of the observed group may possibly explain the fact that, throughout the years, no clear trend in the application rate has been observed. Furthermore, the findings from the logistic regression analyses revealed that the duration of the patient’s stay had a significant, modest effect on the overall NPI acceptance. In the present study, a one-day increase in a patient’s stay led to a high probability of application. This observed association likely reflects both logistical and clinical factors and warrants further investigation in future prospective studies. Clinically, the patients from our study who applied NPIs may have undergone more complex surgical procedures or experienced complications requiring prolonged recovery, making them more eligible or in need of supportive therapies. Thus, patients undergoing complex surgeries, such as reconstructive procedures in plastic surgery, may require extensive postoperative care, including NPIs for pain management, rehabilitation, and emotional support [14]. Additionally, patients with longer stays may develop stronger therapeutic relationships with caregivers, potentially increasing openness to or awareness of NPIs. This could be due to the fact that the longer the patient’s stay is, the greater the possibility of the application of multiple therapy units [15]. In addition, a higher application of stationary NPIs and longer hospital stays may often be driven by the need for comprehensive, holistic care that addresses the multifaceted aspects of patient recovery. Emotional and psychological support through psychotherapy is crucial for many patients, especially those undergoing aesthetic or reconstructive surgeries that impact body image and self-esteem. Extended inpatient care can provide the necessary environment for ongoing psychological support [16]. Our results are supported by evidence from a retrospective study at UCLA, where integrative medicine consults, including acupuncture and trigger-point injections, were more common among patients with extended hospital stays [17]. While integrative oncological and surgical approaches may initially involve longer hospital stays due to the inclusion of NPIs, this early investment in comprehensive care may yield long-term benefits. By addressing pain, anxiety, and functional recovery through therapies such as guided imagery, psychotherapy, and physiotherapy, integrative models may help reduce postoperative complications and subsequent healthcare utilization. This perspective is supported by recent findings from a meta-analysis of psychological prehabilitation, which demonstrated that early mind–body interventions significantly reduced the length of stay, postoperative pain, anxiety, and depression across surgical populations [18]. Several other findings illustrate that integrative therapies can improve perioperative outcomes. A randomized controlled trial in orthopedic patients found that daily post-operative music therapy significantly reduced same-day pain, anxiety, and mood disturbances [19]. Moreover, a meta-analysis of perioperative music interventions demonstrated consistent reductions in anxiety and pain across various adult surgical populations [20]. In contrast, systematic data on NPI use in plastic surgery are scarce. Our study fills this gap by descriptively evaluating six years of real-world integrative practice in a plastic surgery inpatient cohort.

It is important to emphasize that NPIs are not intended as alternatives to surgical treatment but rather as adjuncts aimed at enhancing recovery, emotional well-being, and patient satisfaction. Their integration reflects a holistic approach increasingly valued by patients and supported by emerging clinical models. Physiotherapy, rhythmical embrocations, compresses, and psychotherapy were the most administered NPIs in our cohort. These therapies may play an integral role in enhancing the overall surgical experience and improving outcomes for plastic surgery patients. They have been shown to be effective in promoting physical and mental recovery [21], the management of pain [22,23], the provision of emotional support, and alignment with patient-centered care principles such as anthroposophic medicine [21,24]. Additionally, many patients undergoing plastic surgery expect and value comprehensive care that includes NPIs. These therapies enhance patient satisfaction by addressing holistic health needs and improving overall outcomes beyond the contribution to physical recovery [22].

### Limitations of the Study

This study has several limitations. First, while it presents a descriptive overview of NPI use, it does not assess patient subgroups or clinical outcomes such as pain control, postoperative recovery, medication use, or quality of life. Second, we did not capture reasons for patient refusal or acceptance of NPIs, nor the precise timing of their administration. Third, the relatively small sample size and single-center nature may limit generalizability, particularly as the cohort is heavily weighted toward reconstructive surgery. In addition, a potential selection bias exists due to the inclusion criterion of a minimum five-day hospital stay, which may exclude patients undergoing shorter, less complex procedures and, therefore, underrepresent their attitudes toward NPIs. Finally, our study is limited in that the number of NPI units was not recorded for all observed patients, leading to the necessity for estimating a coefficient and thus introducing potential systematic biases. In addition, several variables were not documented, potentially influencing the study’s measurement validity and liability, and missing associated variables. However, this is one of the first comprehensive studies that systematically analyzes applied integrative anthroposophical NPIs and their association factors in patients receiving plastic surgical procedures between 2016 and 2021 in a German hospital.

## 5. Conclusions

While there is growing interest and patient demand for integrative therapies in plastic surgery, significant gaps in research and practice persist, highlighting the need for more rigorous investigation and integration into clinical care. This exploratory analysis offers a first step in understanding how integrative therapies are applied in the context of plastic surgery within a German hospital setting. While NPIs appear to be well accepted among a subset of patients, further prospective studies are required to evaluate the impact of NPIs on postoperative recovery metrics, including pain management, the duration of the hospital stay, functional rehabilitation, patient-reported quality of life, and the overall satisfaction with care in plastic surgical populations.

## Figures and Tables

**Table 1:** Baseline characteristics of patients.

	*n*
Number of patients, *n* (%)	265 (100)
Age, years, median (IQR)	65 (52–80)
Gender	
Male, *n* (%)	115 (43.3)
Female, *n* (%)	150 (56.6)
Stay duration, days, median (IQR)	12 (7–8)

Baseline characteristics: *n*, number; %, percent; *n* = 265.

**Table 2:** Types of plastic surgery.

Types	Number of Patients (*n* = 265)	Percent %
Reconstructive surgery ^1^, *n* (%)	240 ^1^	90.6
Hand surgery, *n* (%)	15	5.7
Aesthetic surgery, *n* (%)	7	2.6
Oncological surgery, *n* (%)	1	0.4
Burn surgery ^2^, *n* (%)	2 ^2^	0.8

The percentage of plastic surgery types does not necessarily add to 100% as patients may have received multiple types of surgery. *n*, number; %, percent; *n* = 265; ^1^ including reconstructive aesthetic surgery; ^2^ including reconstructive burn surgery.

**Table 3:** Application of NPIs.

Year of Patient’s Admittance to Hospital	Patients per Year (Stay in Hospital > 5 Days)	Patients Receiving NPIs
2016	21	9 (42.9)
2017	46	26 (56.5)
2018	35	8 (22.9)
2019	70	26 (37.1)
2020	42	30 (71.4)
2021	51	14 (27.5)
**Total**	**265**	**113 (42.6)**

NPIs, non-pharmacological interventions.

## Data Availability

The datasets that support the findings in this article are not publicly available for privacy and security reasons but can be obtained from the corresponding author upon reasonable request.
